# Autonomous conversational agents for loneliness, social isolation, depression, and anxiety in older people without cognitive impairment: Systematic review and meta-analysis

**DOI:** 10.1017/S0033291725103073

**Published:** 2026-01-20

**Authors:** Yuto Satake, Harry Costello, Nimesh Naran, Daiki Ishimaru, Manabu Ikeda, Robert Howard

**Affiliations:** 1 Division of Psychiatry, University College London, London, UK; 2 Department of Psychiatry, The University of Osaka Graduate School of Medicine, Suita, Japan; 3 North London NHS Foundation Trust, London, UK; 4 Department of Medical Technology, The University of Osaka Hospital, Suita, Japan

**Keywords:** chatbot, loneliness, mental health, personal voice assistant, robot

## Abstract

Loneliness is a major psychological challenge in older adulthood, contributing to increased risks of depression, anxiety, and mortality. Conversational agents – technologies that interact with users via natural language – have emerged as potential tools to support psychological well-being in later life. This systematic review and meta-analysis evaluated the effects of autonomous conversational agents, including robotic and nonrobotic systems, on loneliness, as well as social isolation, depression, and anxiety in older people without cognitive impairment. Seventeen studies with pre–post intervention data were included. Nine used physically embodied robots and eight employed nonrobotic agents, such as personal voice assistants, chatbots, or screen-based embodied agents. Due to the limited number of high-quality comparison studies, all meta-analyses were based on within-group pre–post comparisons. Meta-analytic results showed mild to moderate improvements in loneliness (standardized mean changes using change score [SMCC] = 0.350, 95% confidence interval [CI]: 0.180–0.520) and depression (SMCC = 0.464, 95% CI: 0.327–0.602), with no study reporting symptom worsening. No study included validated measures of social isolation, and only one assessed anxiety. These findings indicate that conversational agents may offer scalable support for older adults’ mental health, with potential especially for reducing loneliness and depression. Nonetheless, methodological limitations, including lack of blinded outcome assessment, inconsistent reporting, and heterogeneous intervention designs, underscore the need for more rigorous research. Advances in large language models may further enhance the responsiveness and relevance of these technologies for supporting psychological well-being in aging populations.

## Introduction

Loneliness has been associated with increased risks of mortality and mental health conditions, such as depression and anxiety (Holt-Lunstad, 2024; Lee et al., 2021; Santini et al., 2020). With advancing age, experience of bereavement, declining health, and reduced income may contribute to fewer opportunities for social interaction, leading to increased feelings of isolation (Luhmann & Hawkley, 2016). Globally, approximately one in four older people experience social isolation (Teo, Cheng, Cheng, Lau, & Lau, 2023), and more than 20% live alone in many countries (Japan Cabinet Office, 2024; United Nations, 2020). In response to these concerns, several governments have implemented formal initiatives, such as the appointment of Ministers for Loneliness in the United Kingdom and Japan, and the World Health Organization established a Commission on Social Connection in 2023 (World Health Organization, 2024). These developments underscore the urgent need for scalable and accessible interventions to reduce loneliness and support mental well-being in later life.

Against this backdrop, conversational agents – systems that interact with users through natural language – have attracted increasing attention (Döring et al., 2022; Teo, Yoong, Chan, & Jiang, 2025). A wide range of technologies has emerged, including social robots, personal voice assistants (PVAs), chatbots, and screen-based embodied agents. These tools hold potential for alleviating loneliness and fostering emotional connection, especially among socially isolated older adults. Recent advances in large language models (LLMs) have dramatically expanded the capabilities of conversational agents as a flexible and empathetic talking partner (Guo et al., 2023; Welivita & Pu, 2024). While limitations such as delayed system responses and constrained conversational quality persist, and ethical concerns – including privacy and misinformation – remain, both domains are the focus of ongoing technological development and interdisciplinary debate (Irfan, Kuoppamäki, Hosseini, & Skantze, 2025).

Although information-technology interventions (Balki, Hayes, & Holland, 2022), social robots (Gasteiger, Loveys, Law, & Broadbent, 2021; Pu, Moyle, Jones, & Todorovic, 2019; Yu, Sommerlad, Sakure, & Livingston, 2022), and chatbots (Zhang, Wong, & Bayuo, 2024) have been reviewed in the context of older-adult care, a comprehensive synthesis that spans both robotic and nonrobotic autonomous conversational agents is still lacking. This gap complicates the design of next-generation, LLM-enabled agents aimed at reducing loneliness in community-dwelling older adults without dementia. Therefore, we conducted a systematic review and meta-analysis to quantify the effects of autonomous conversational agents on loneliness and, in addition, on other psychological outcomes that might plausibly benefit from such interventions – namely depression, social isolation, and anxiety – in older adults without cognitive impairment.

## Methods

### Study design

Systematic review and meta-analysis conducted in accordance with the Preferred Reporting Items for Systematic Reviews and Meta-Analyses (PRISMA) 2020 guidelines. The review aims to synthesize the evidence regarding the efficacy of autonomous conversational agents on loneliness, social isolation, depression, and anxiety in older adults without cognitive impairment.

### Protocol and registration

The protocol for this review was prospectively registered in the International Prospective Register of Systematic Reviews under the registration number (CRD42024605387) on 31 October 2024.

### Eligibility criteria

Inclusion criteria: (1) Participants aged 60 years or older without cognitive impairment or dementia. (2) Use of autonomous conversational agents (e.g. social robots, chatbots, and embodied conversational agents). (3) Reported validated quantitative measures of loneliness, social isolation, depression, and/or anxiety both before and after the intervention. (4) Any type of intervention study, including randomized controlled trials, nonrandomized controlled studies, and single-group pre–post designs.

Exclusion criteria: (1) Not peer-reviewed. (2) Published in a language other than English or Japanese. (3) Gray literature, defined as non–peer-reviewed sources, such as unpublished reports, theses, and conference abstracts. Peer-reviewed conference papers (particularly from engineering fields) were included if they met all other inclusion criteria.

Although our initial eligibility criterion specified participants aged 60 years or older, we found that some studies targeting older adults also included participants under the age of 60 years. To accommodate these real-world recruitment practices while maintaining our focus on older populations, we included studies in which the mean participant age was 60 years or older. In cases where essential outcome data were not reported in the published articles, we contacted the authors to obtain the necessary information and included the study whenever possible based on the additional data provided.

### Information sources and search strategy

A comprehensive search was conducted using the following electronic databases: Ovid MEDLINE (ALL), Embase (1974–2024), APA PsycINFO, CINAHL Plus (EBSCOhost), Web of Science, IEEE Xplore, ACM Digital Library, Ichushi-Web, CiNii Research, and the National Diet Library. The search included studies published up to 1 November 2024.

For MEDLINE, Embase, PsycINFO, CINAHL Plus, and Web of Science, we performed title, abstract, and keyword searches. For IEEE Xplore, we conducted title and abstract searches. For the remaining databases, we conducted searches without applying filters.

The search terms included Population terms (e.g. ‘aged’ and ‘elder*’), Technology terms (e.g. ‘chatbot*’, ‘robot*’, and ‘conversation* agent’), and Outcome terms (e.g. ‘lonel*’ and ‘depress*’). Further details of the search strategy are presented in the Supplementary Note. In addition, relevant review articles were manually screened to identify additional studies.

### Selection process

Initial citation search and the collection of title/abstract were conducted by YS. Title/abstract and full-text screenings were performed independently by two reviewers. For English-language articles, the screening was conducted independently by YS and NN. For Japanese-language articles, the screening was independently conducted by YS and DI. Discrepancies at any stage were resolved through discussion among four reviewers (HC, YS, DI, and NN).

### Data collection process

YS imported all retrieved citations into a citation manager (Zotero), and duplicates were removed. Full texts of potentially relevant studies were assessed in detail. Data extraction was performed by YS using a predefined spreadsheet and included study characteristics, participant demographics, intervention details, outcome measures, and pre–post change scores. DI and NN cross-checked the spreadsheet.

### Data items

The following data were extracted: study design, number of participants per group, participant characteristics, intervention type and duration, outcome measures for loneliness, social isolation, depression, and anxiety, and corresponding pre- and post-intervention scores.

### Study risk of bias assessment

Two reviewers (YS and DI) independently assessed the risk of bias of each included study. Although we initially planned to use Version 2 of the Cochrane Risk of Bias tool for randomized controlled trials and the Newcastle-Ottawa Scale (NOS) for nonrandomized studies, the number of randomized controlled trials with two-group comparisons was too limited. Therefore, NOS was applied to all included studies regardless of study design. Any discrepancies between reviewers were resolved through discussion among the review team.

### Effect measures

We had planned to use standardized mean differences to compare intervention and control groups. However, due to the limited number of studies with two-group comparisons, we calculated effect sizes within each intervention group using the standardized mean change using change score (SMCC) approach. The primary effect measure was SMCC based on validated scales of loneliness. Additional outcomes included SMCC values for social isolation, depression, and anxiety, where data were available. Since none of the included studies reported pre–post correlations required for SMCC calculations, we assumed a correlation coefficient (*r*) of 0.5 as a conservative default, following common practice in meta-analytic research.

### Synthesis methods

We conducted meta-analyses using a random-effects model if three or more eligible studies reported on the same outcome. Meta-analyses were performed using R software with the metafor package. We assessed statistical heterogeneity using the *I* ^2^ statistic and explored sources of heterogeneity where appropriate. To investigate potential sources of heterogeneity, we conducted exploratory subgroup analyses based on the type of conversational agent used. Specifically, we compared outcomes from studies employing physically embodied robots (e.g. humanoid social robots) with those using nonrobotic conversational agents (e.g. text-based chatbots or screen-based avatars). For contextualization of clinical relevance, we additionally summarized the raw pre–post change on the Geriatric Depression Scale (GDS), the most frequently used validated scale, across eligible studies as a simple unweighted mean. Studies with reporting inconsistencies were excluded from this descriptive summary. This analysis was exploratory and not part of the inferential meta-analyses.

In addition, we performed leave-one-out (LOO) analyses and excluded small-sample studies to explore their influence on overall results. As part of the sensitivity analyses related to the assumed correlation coefficient (*r*), we re-ran all meta-analyses using *r* = 0.3 and *r* = 0.7 to evaluate the impact of this assumption on the estimated effect sizes.

### Reporting bias assessment

Funnel plots were generated, and Egger’s test was performed to assess potential publication bias. If asymmetry was observed, we planned to conduct sensitivity analyses and interpret the results with caution, considering possible small-study effects.

### Results

### Study selection

**Figure 1.**
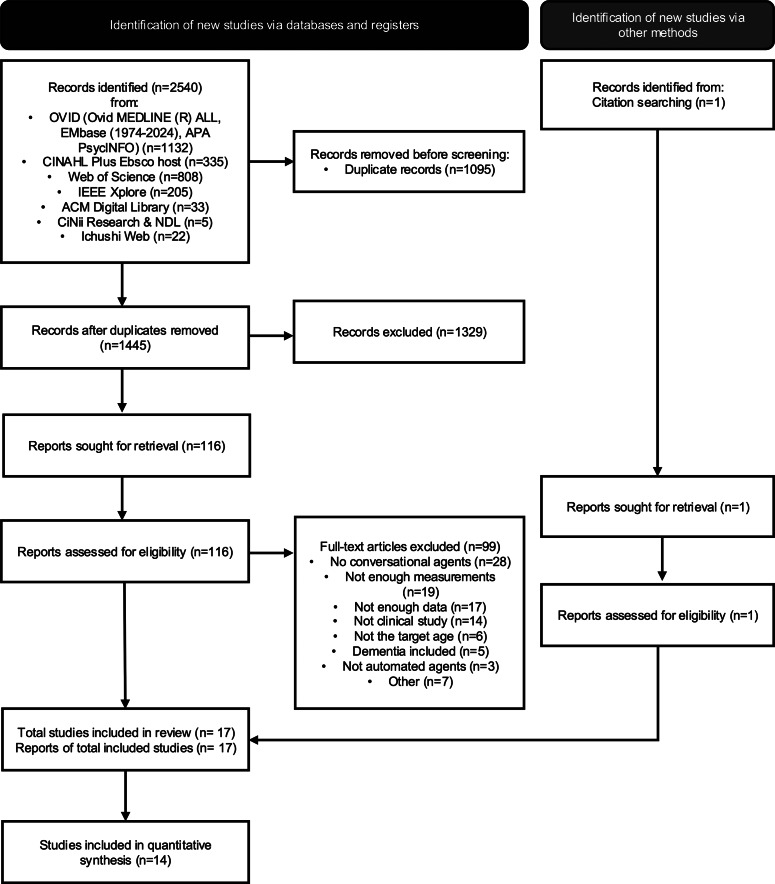
PRISMA 2020 flow diagram.

A total of 2,540 records were identified through database searches. After removing duplicates, 1,445 unique records remained. Of these, 116 full-text articles were assessed for eligibility, and 17 studies met the inclusion criteria. Three of these were excluded from the effect size calculation due to missing group-level means (Broadbent et al., 2014; Ring, Shi, Totzke, & Bickmore, 2015; Wang & Li, 2024). The study selection process is illustrated in the PRISMA 2020 flow diagram (Figure 1).

### Study characteristics

Study characteristics are summarized in Table 1, with further details provided in Supplementary Table S1. Sample sizes of the intervention groups ranged from 4 to 291 participants (median = 18; mean = 43.6; standard deviation [SD] = 74.1). Participants’ ages ranged from 50 to 98 years, with most studies targeting adults aged 65 years or older. Based on data from 16 of the 17 included studies, other than one without a reported mean age, the weighted mean age of participants across intervention groups was 78.1 years. SDs were available for 14 studies, and a pooled SD was therefore not calculated. The majority of participants were women, and three studies included only female participants. Of the 17 included studies, 10 specifically targeted community-dwelling older adults living alone. Two studies involved community-dwelling older people without restricting to living situation. The remaining five studies were conducted in institutional or assisted living settings, including nursing homes, care homes, and retirement villages.

**Table 1.** The summary of the included studies

Author (year)	Country	N	Living situation	Diagnosis	Intervention type	Agent description	Duration/Frequency	Outcomes	Summary
Yan et al. (2024)	USA	15	Living alone (independent, at home)	Community-dwelling older adults	PVA	Alexa (Amazon Echo: A commercial voice-controlled smart speaker equipped)	4 weeks; daily use; ≥5 times/day (instructed); reminders sent on days 7, 14, and 21 if below threshold	Lo	・Significant reduction in loneliness after 4 weeks ・Total time spent with Alexa was significantly correlated with loneliness reduction
Wang and Li (2024)	China	4	Nursing home residents	NR; assumed cognitively normal nursing home residents	Chatbot	ChatGPT 3.0 (via headset + screen + researcher-supported dialogue)	8 weeks; 15–30 min, once/week (8 sessions total)	Lo, De	・No significant changes in loneliness and depression
Lee et al. (2024)	USA	30	70% lived alone	Community-dwelling older adults	Robot	Hyodol: Doll-shaped socially assistive humanoid robot with speech	4 months; daily use; individual usage not strictly prescribed	Lo, De	・Depressive symptoms improved significantly ・Loneliness decreased but not significantly
Jones et al. (2024)	USA	34	All participants lived alone in independent living facilities	Community-dwelling older adults; Cognitively normal to mildly impaired (MoCA mean = 23.5)	PVA	Alexa (audio-only, Echo Dot vs. video, Echo Show)	12 weeks; daily use; ≥30 min/day (prescribed routine activities)	Lo	・Significant reduction in loneliness ・Significant increase in perceived social support
Chou et al. (2024)	Taiwan	15	47% living alone	Psychiatric outpatients with depression or anxiety disorders	Chatbot	LINE-based health chatbot	4 weeks; daily use; 82.5% adherence (≥65 group)	Lo, De, An	・Significant loneliness reduction ・No significant change in depression or anxiety
Lim (2023)	South Korea	31	Living alone	Community-dwelling older adults	Robot	PIO: Parrot-shaped socially assistive robot	6 weeks; 50 min, 2 sessions/week (12 sessions total)	Lo, De	・Significant improvement in loneliness, depression, and cognitive function
Lee, Nam, Chon, Park, and Choi (2023)	South Korea	169	95.3% lived alone; 39% had no contact with children	Community-dwelling older adults	Robot	Hyodol: Doll-shaped socially assistive humanoid robot with speech	3 months (6 months also); daily use; 24 h inactivity triggers alert	De	・Depressive symptoms significantly improved at 3 months, but the effect was not maintained at 6 months.
Park and Kim (2022)	South Korea	291	Living alone	Community-dwelling older adults; multiple chronic illnesses are common	PVA	NUGU Candle: Commercial Korean AI speaker with voice interaction features	2 months; daily use; comparison between frequent (≥5/week) and intermittent users	Lo, De	・Significant reduction in loneliness and depression ・Loneliness reduction more pronounced in frequent users
Papadopoulos et al. (2022)	UK and Japan	12	Care home residents living in a single room	Older adults without severe dementia, severe mental health issues, or severe frailty	Robot	Pepper: Socially assistive humanoid robot with speech. It was culturally personalized (Experimental group). 4 feet tall	2 weeks; 6 sessions × up to 3 h each	Lo	・No significant change in loneliness
Kramer, Van Velsen, Clark, Mulder, and De Vet (2022)	The Netherlands	32	Living alone	Community-dwelling older adults	Screen-based embodied agents	PACO: Two 2D cartoon-style avatars (Herman for nutrition, Ellen for social advice), text-based, non-animated.	8 weeks; daily use; self-paced use; median total use 15 h	Lo	・No significant change in loneliness and eating behavior
Jones et al. (2021)	USA	16	Living alone	Community-dwelling older adults aged 75+ years with normal cognitive functioning	PVA	Alexa (Amazon Echo: A commercial voice-controlled smart speaker equipped)	4 weeks (8 weeks actually, but it was not for measurements of loneliness); daily; minimum 5 interactions per day during the first 4 weeks (mean ~ 18/day); voluntary use thereafter	Lo	・Significant reduction in loneliness ・Relational greetings to Alexa predicted loneliness reduction
Kargar et al. (2017)	USA	7	Living at an assisted living facility	Older adults with no to moderate depression	Robot	eBEAR: Animal-like social robot with facial expression generation and dialogue capabilities	1 month; 3 sessions per week (up to 1 h/session)	De	・Improvement in depression scores in several participants
Broadbent et al. (2016)	New Zealand	27	Living in rest home and nursing home units	Older adults; mixed cognitive status (35% with AMTS ≤6, suggesting cognitive impairment)	Robot	Guide: Stationary touchscreen robot offering scripted speech interactions. Cafero: Stationary touchscreen robot with similar functions to Guide.	12 weeks; robots were available for 14 h (6 AM–8 PM) a day at the lounge of facilities; no usage requirement enforced	De	・No significant differences in depression, quality of life, or mobility between groups
Ring et al. (2015)	USA	7	Living alone	Older adults without significant depression symptoms (screened with PHQ–2)	Screen-based embodied agents (proactive version)	Animated touchscreen-based avatar using synthetic voice, facial expressions, and gestures	1 week; daily; proactive version initiated conversation via motion sensor	Lo	・Proactive agent significantly reduced loneliness and increased happiness and comfort compared to the passive agent ・Longer interaction time correlated with greater loneliness reduction
Broadbent et al. (2014)	New Zealand	29	Independent living apartments in a retirement village	Older adults; cognitive function assessed (AMTS mean = 9.2/10); mostly cognitively intact	Robot	iRobiQ: Small table-top robot with touchscreen. Cafero: Larger robot with touchscreen	6 weeks robot condition +6 weeks no-robot control (crossover), with an 18-day washout period; daily; no enforced usage amount	De	・No significant improvements in depression, quality of life, or medication adherence detected
Tanaka et al. (2012)	Japan	18	Living alone	Community-dwelling older female adults without diagnosed dementia (MMSE ≥24)	Robot	Kabochan: Doll-shaped socially assistive humanoid robot with speech	8 weeks; daily; interactions not strictly standardized	De	・The change of depression scale was not statistically significant. ・Cognitive scales showed improvements.
Suzuki, Kanamori, and Ueda (2005)	Japan	4	Living alone	Community-dwelling older female adults without diagnosed dementia	Robot	Ifbot; humanoid robot capable of recognizing speech and displaying emotional expressions	4 weeks; daily; encouraged to interact daily	De, Lo	・Some participants showed improvement in depression scores

*Note*: *N* and Diagnosis represent the number of participants and diagnoses in the intervention group. Abbreviations: PVA, personal voice assistant; Lo, loneliness; De, depression; An, anxiety; NR, not reported; MoCA, Montreal Cognitive Assessment; MMSE, Mini-Mental State Examination; PHQ-2, Patient Health Questionnaire-2; AMTS, Abbreviated Mental Test Score.

The most frequently reported additional outcome was quality of life, included in seven studies. Study designs included eight single-arm pre–post studies (including one case series), four nonrandomized two-arm studies, and three randomized controlled trials – one of which employed a crossover design and another a delayed intervention model. Of the 17 studies, 12 assessed loneliness, 11 assessed depression, one assessed anxiety, and none assessed social isolation using validated outcome measures. Ten of 11 studies evaluating depression used the GDS (Yesavage et al., 1982), and 1 used the Patient Health Questionnaire-9 (Kroenke, Spitzer, & Williams, 2001). Ten of 12 studies evaluating loneliness used the UCLA Loneliness Scale (in various versions) (Hays & DiMatteo, 1987; Russell, Peplau, & Cutrona, 1980; Russell, 1996), 1 used De Jong Gierveld Loneliness Scale (Gierveld & Tilburg, 2006), and 1 used Ando, Osada and Kodama Loneliness Scale (Ando, Hisao, & Kodama, 2000).

Interventions varied widely in both platform and function. Nine deployed robots, while eight utilized nonrobotic conversational agents: four involved PVAs, two used screen-based embodied agents, and two used chatbots. The most commonly used device was the Amazon Echo (three studies) (Jones et al., 2024; Jones et al., 2021; Yan et al., 2024), followed by Hyodol (two studies) (Lee, Nah, Kim, Choi, & Park, 2024; Lee et al., 2023). Only one study deployed LLM-based conversational agents (Wang & Li, 2024). All other studies used different devices or applications. Agent functions ranged from conversation-based interaction to features such as riddles, online shopping support, reminders, games, video calling (e.g. Skype), encouragement for physical activity, singing, dancing, dietary suggestions, and cognitive training. Intervention duration ranged from three brief sessions to 4 months, with usage intensity varying from short daily interactions to continuous availability.

### Risk of bias in studies

The mean NOS score was 4.1 (SD = 1.2), with a range of 2–7. One study was rated as high quality (7–9 points), 10 studies as moderate quality (4–6 points), and 6 studies as low quality (0–3 points) (Table 2). Notably, some NOS items – such as ‘Selection of non-exposed cohort’ and ‘Comparability of cohorts’ – were rated as ‘not applicable’ for single-arm designs. Importantly, no study received a star for ‘Assessment of outcome’, reflecting the absence of blinded outcome evaluation. While most studies received stars for ‘Ascertainment of exposure’ and ‘Was follow-up long enough for outcomes to occur?’, the actual quality of implementation in these items varied. Although not formally assessed within the NOS framework, several two-arm studies included participants with notably high educational backgrounds in the intervention groups, which is one of the essential selection biases (Lim, 2023; Papadopoulos et al., 2022; Wang & Li, 2024).

**Table 2.** Risk of bias assessment using the Newcastle-Ottawa Scale

Author (Year)	Total score (max 9 ☆)	Selection (max 4 ☆)				Comparability (max 2 ☆)	Outcome (max 3 ☆)		
		Representativeness of the exposed cohort	Selection of the non-exposed cohort	Ascertainment of exposure	Demonstration that the outcome of interest was not present at the start of the study	Comparability of cohorts based on the design or analysis	Assessment of outcome	Was the follow-up long enough for outcomes to occur	Adequacy of follow-up of cohorts
Yan, Johnson, and Jones (2024)	4	☆	NA	☆	☆	NA		☆	
Wang & Li, (2024)	3		☆	☆				☆	
Lee et al. (2024)	5	☆	NA	☆	☆	NA		☆	☆
Jones et al. (2024)	4	☆	NA	☆	☆	NA		☆	
Chou et al. (2024)	3	☆	NA	☆		NA		☆	
Lim (2023)	6	☆	☆	☆	☆			☆	☆
Lee et al. (2023)	5	☆	NA	☆	☆	NA		☆	☆
Park and Kim (2022)	5	☆	NA	☆	☆	NA		☆	☆
Papadopoulos et al. (2022)	4		☆	☆		☆☆			
Kramer et al. (2022)	4	☆	NA	☆	☆	NA		☆	
Jones et al. (2021)	3	☆	NA	☆		NA		☆	
Kargar et al. (2017)	3	☆	NA	☆		NA		☆	
Broadbent et al. (2016)	2		☆					☆	
Ring et al. (2015)	5	☆	☆	☆	☆				☆
Broadbent et al. (2014)	3		☆	☆				☆	
Tanaka et al. (2012)	7	☆	☆	☆	☆	☆		☆	☆
Suzuki et al. (2005)	4	☆	NA	☆	☆	NA		☆	

☆ indicates that the study met the criterion and was awarded 1 point on the Newcastle–Ottawa Scale (NOS); ☆☆ indicates 2 points where applicable (comparability). Blank cells indicate 0 points; NA = not applicable. Total scores were calculated by summing awarded stars.

### Results of individual studies

**Figure 2.**
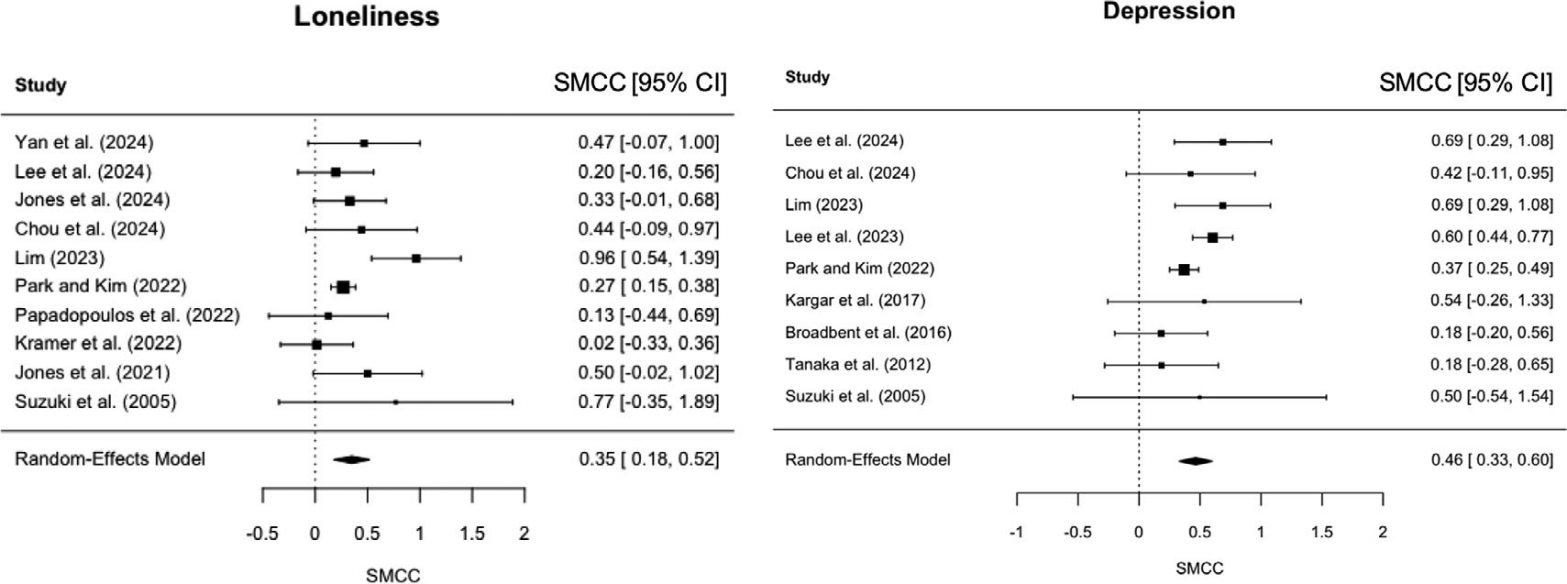
Forest plots of pooled standardized mean change using change score (SMCC). Each study’s point estimate and 95% CI are shown. The left panel shows results for loneliness, and the right panel shows results for depression. SMCC was calculated by dividing the mean pre–post difference by the SD of the change scores, assuming a pre–post correlation of *r* = 0.5. Positive values indicate improvements (i.e. reductions in loneliness or depressive symptoms) following the intervention. Pooled effect sizes were estimated using a random-effects model.

Among the included studies, 10 assessed loneliness and nine assessed depression using validated pre- and post-intervention measures in the intervention group. The effect sizes and their 95% confidence intervals (CIs) for each individual study are presented in Figure 2. For loneliness, SMCCs ranged from 0.02 to 0.96, and for depression, they ranged from 0.18 to 0.69. Notably, no study showed a negative effect (i.e. worsening of symptoms). Anxiety was evaluated in only one study using a chatbot intervention (Chou, Lin, Lee, Lee, & Cheng, 2024), which reported no significant pre–post change in anxiety with a SMCC score of 0.23 (95% CI [0.10–0.56]), suggesting a reduction in anxiety symptoms. Although a study measured a subjective measure of social support (Jones et al., 2024), no studies deployed an objective scale for social isolation.

### Results of syntheses

Meta-analyses were conducted for loneliness and depression only based on the number of included papers.

Ten studies were included in the meta-analysis for loneliness. The pooled SMCC was 0.350 (95% CI: 0.180–0.520, *p* < 0.001), indicating a mild to moderate reduction in loneliness following interventions with conversational agents (Figure 2). Statistical heterogeneity was moderate (*I* ^2^ = 46.8%, *τ* ^2^ = 0.029), and the heterogeneity test was not statistically significant (*Q*(9) = 14.82, *p* = 0.096). The exploratory subgroup analyses comparing studies using robotic agents (*k* = 4) versus nonrobotic agents (*k* = 6) showed a somewhat larger pooled effect in the robotic group (0.479, 95% CI: 0.023–0.935, *p* = 0.039, *I* ^2^ = 65.5%) compared to the nonrobotic group (0.274, 95% CI: 0.174–0.374, *p* < 0.001, *I* ^2^ = 0%) (Supplementary Figure S1).

Nine studies were included in the meta-analysis for depression. The pooled SMCC was 0.464 (95% CI: 0.327–0.602, *p* < 0.001), indicating a mild to moderate reduction in depressive symptoms following the intervention (Figure 2). Heterogeneity was low to moderate (*I* ^2^ = 35.9%, *τ* ^2^ = 0.013), and the test for heterogeneity was not statistically significant (*Q*(8) = 11.14, *p* = 0.194). Across studies using the GDS-15 with valid reporting (*n* = 7; excluding Lim, 2023 due to reporting inconsistencies), the mean pre–post raw change was 1.3 points (SD = 0.6). Subgroup analysis was not performed for depression due to the limited number of studies using nonrobotic agents (*n* = 2).

LOO analyses were conducted for both loneliness and depression to assess the robustness of the findings. In both cases, exclusion of individual studies did not materially alter the overall pooled effect sizes, indicating that no single study unduly influenced the results (Supplementary Figure S2). Notably, Jones et al. (2024) was the only meta-analyzed study that included any participants aged <60 years, and its exclusion produced results very similar to the main analyses (Jones et al., 2024). Ring et al. (2015) also included some participants aged <60 years, but was not included in the meta-analyses due to missing group-level means (Ring et al., 2015). Additionally, sensitivity analyses were performed using alternative assumptions for the pre–post correlation used in the SMCC calculation. The primary analysis used *r* = 0.5, but analyses were also run using *r* = 0.3 and *r* = 0.7. The pooled effect sizes remained stable across these values, suggesting that the results were not sensitive to variations in the assumed correlation (Supplementary Figures S3 and S4).

### Reporting biases

To assess potential reporting biases, funnel plots were visually inspected for asymmetry in the meta-analyses of loneliness and depression. As shown in Supplementary Figure S5, the distribution of effect sizes appeared generally symmetrical for both outcomes, suggesting a low risk of publication bias. We also conducted Egger’s regression test to statistically assess funnel plot asymmetry. The test indicated no significant evidence of small-study effects for either outcome: loneliness (*z* = 1.0, *p* = 0.295) and depression (*z* = −0.04, *p* = 0.967). These findings support the visual interpretation that the risk of publication bias was low for both outcomes.

## Discussion

This systematic review and meta-analysis included 17 studies evaluating the effects of autonomous conversational agents on loneliness, depression, social isolation, and anxiety in older adults without cognitive impairment. Although several studies included a control group, only six provided sufficient data for group-level comparisons, and all meta-analytic effect sizes were therefore based on pre–post changes within intervention groups. Meta-analytic results indicated small to moderate improvements in loneliness (SMCC = 0.350) and depressive symptoms (SMCC = 0.464), with no evidence of symptom worsening in any study. Subgroup analyses for loneliness suggested that physically embodied social robots may yield greater benefits than nonrobotic agents, though the small number of robot-based studies merits caution. Sensitivity analyses confirmed the robustness of findings, and no significant publication bias was detected. These results support the potential utility of conversational agents, particularly social robots, in promoting psychological well-being among older adults without cognitive impairment.

This review found that research on conversational agents for cognitively unimpaired older adults has primarily focused on loneliness and depression, with anxiety and social isolation rarely addressed. Only one study assessed anxiety (Chou et al., 2024), and none included validated measures of social isolation. This is somewhat surprising, given that both are well-documented challenges in later life (Holt-Lunstad, 2024) and often examined in conjunction with loneliness in related research fields (Gadbois et al., 2022; Kwok et al., 2024; Lai, Li, Ou, & Li, 2020). The absence of social isolation measures may reflect the dominance of self-reported psychological constructs in this field or a lack of consensus on how to operationalize social connectivity objectively. The consistent use of the UCLA Loneliness Scales (in various versions) and the GDS suggests a preference for well-validated, standardized tools in this literature. However, inconsistencies in the reported score ranges – where some outcomes deviated from the expected theoretical limits of the scales – were occasionally observed, raising concerns about scoring accuracy or reporting clarity in a few studies.

The findings of this review suggest that conversational agents, including social robots and voice assistants, may help alleviate loneliness and depressive symptoms in older adults. To our knowledge, no previous meta-analysis has specifically focused on conversational agents in this population, and only a limited number of reviews have addressed the topic. For example, recent scoping and systematic reviews have reported potential benefits of PVAs and screen-based embodied agents for loneliness and depression among older adults, although the evidence remains preliminary (Castro Martínez, Encuentra, Eulàlia, & Pousada Fernández, 2025; Teo et al., 2025). One meta-analysis found a moderate effect of relational agents – including nonconversational robots – on loneliness (Sha et al., 2024). Other reviews have also reported promising effects of social robots on psychological outcomes in older populations (Chen, Jones, & Moyle, 2018; Yen, Huang, Chiu, & Jin, 2024). By contrast, some reviews have raised concerns about the quality and quantity of available evidence, concluding that the effectiveness of social robots remains uncertain (Pu et al., 2019; Yu et al., 2022). These concerns also apply to the present review. None of the 17 included studies implemented blinded outcome assessment, indicating a potential risk of detection bias. Although several studies included comparison groups, only five were able to be meta-analyzed as a comparison study, and their methodological quality was limited. Therefore, we opted to analyze within-group changes using a single-arm pre–post design to explore the psychological potential of conversational agents in cognitively unimpaired older adults. The diversity in intervention duration, intensity, and follow-up periods, along with inconsistencies in reporting dropout rates, highlights the need for greater methodological standardization to improve future comparability and cumulative synthesis. Moreover, while pooled standardized effects were small to moderate for depression, the absolute mean change on the GDS-15 was modest on average. While a universally accepted minimal clinically important difference for the GDS-15 is lacking, a recent individual patient data meta-analysis reported a minimal detectable change at 67% confidence of ~1.95 points (González-Domínguez et al., 2024), suggesting that the average pre–post change observed here (1.3 points) was modest. Given that most studies lacked controls and the few controlled trials showed small between-group differences (Broadbent et al., 2016; Tanaka et al., 2012), the clinical meaningfulness of the pooled within-group estimates should be interpreted with caution.

The exploratory subgroup analyses for loneliness conducted in this review suggested that physically embodied social robots may produce somewhat greater effects compared to nonrobotic conversational agents. According to Weiss, emotional loneliness arises from a lack of companionship, which offers reassurance of worth and an outlet for intimate self-disclosure. It is well established that humans tend to respond to media such as computers and television as if they were social entities (Nass & Moon, 2000; Reeves & Nass, 1996). However, which features of such agents actually foster a sense of companionship remains unclear. Prior studies have indicated that nonverbal features – such as facial expressions, synchronized gestures, and gaze – can enhance the perception of companionship in embodied conversational agents and social robots (Kargar et al., 2017; Ring et al., 2015). Furthermore, in a study using a PVA, participants with higher baseline loneliness scores were more likely to greet the device as they would a person, and such behavior predicted subsequent reductions in loneliness (Jones et al., 2021). Similarly, in a study involving a doll-shaped social robot (Hyodol), participants spontaneously referred to the robot as a ‘grandchild’ or ‘companion’, suggesting that personification may have played a role in enhancing emotional engagement and therapeutic benefit (Lee et al., 2024). It is possible that physical embodiment – through gaze, movement, and affective expression – fosters anthropomorphism and, in turn, strengthens companionship, which may help explain why robots were more effective in alleviating loneliness. However, the interventions included in this review were diverse in both form and function, often combining features such as reminders, music playback, and health-related messages. As such, it remains difficult to isolate which specific elements contributed most to the observed effects. This comparison is hypothesis-generating and should be interpreted with caution due to small numbers and heterogeneity. Future research should aim to disentangle these components and examine the relative impact of conversational flexibility, physical expression, and other design features (Supplementary Figure S6).

This review has several methodological strengths. To our knowledge, it is the first systematic review and meta-analysis to compare the effects of both robotic and nonrobotic conversational agents in older adults. We examined a wide range of outcomes – including loneliness, depression, and anxiety – and also collected data on background characteristics such as living situation and cognitive status. Robustness of the findings was assessed through sensitivity analyses and evaluation of publication bias. However, there are several limitations to consider. First, our pooled estimates rely primarily on within-group pre–post changes in the intervention arms, which are vulnerable to confounding, regression to the mean, and other nonspecific effects. Second, overall study quality was modest: only one study was rated high quality, and many exhibited multiple risks of bias. Third, interventions varied widely in content, agent type, functionality, setting, and usage frequency. This heterogeneity increases statistical uncertainty and precludes firm conclusions about causality or about which components are most effective. Fourth, although we excluded trials designed for populations with diagnosed cognitive disorders, some studies may have enrolled participants with mild cognitive impairment, which could affect responsiveness. Fifth, participant characteristics were inconsistent across studies; several samples were skewed toward individuals with higher educational attainment or socioeconomic status, limiting generalizability. Finally, few studies included long-term follow-up, so the durability of any observed benefits remains uncertain. In light of these issues, our findings should be interpreted as overall trends across diverse implementations, rather than evidence of comparative or component-specific efficacy.

## Conclusion

This systematic review highlights that conversational agents – including both robotic and nonrobotic systems – have the potential to support psychological well-being among community-dwelling older adults without cognitive impairment. Although the overall evidence base remains limited and heterogeneous, particularly in terms of study quality and intervention duration, the findings suggest that such technologies may help alleviate loneliness and depressive symptoms. This trend may be further supported by the development of LLMs. Further high-quality, long-term studies with rigorous designs are needed to clarify their effectiveness and inform future development and implementation.

## Supporting information

10.1017/S0033291725103073.sm001 Satake et al. supplementary material 1 Satake et al. supplementary material

10.1017/S0033291725103073.sm002 Satake et al. supplementary material 2 Satake et al. supplementary material

## Supplementary material

The supplementary material for this article can be found at http://doi.org/10.1017/S0033291725103073.
