# Supplementary material for: Autonomous conversational agents for loneliness, social isolation, depression, and anxiety in older people without cognitive impairment: Systematic review and meta-analysis
Source: Psychol Med. 2026 Jan 20;56:e27. doi: 10.1017/S0033291725103073 (PMC12885342; doi:10.1017/S0033291725103073)
Supplement: Satake et al. supplementary material 2 — Satake et al. supplementary material [file S0033291725103073sup002.docx]

Supplementary Note. Search Terms

Citation Search　1/11/2024

**English Databases**

**・Ovid search**　 Filter: TI/AB/Keyword heading

Medline Ovid MEDLINE (R ) ALL

Embase (1974-2024)

APA PsycINFO

**・CINAHL Plus Ebsco host**　 Filter: TI/AB/MW

**・Web of Science**　 Filter: TI/AB/author keywords

(Search Terms)

"aged" OR "elder*" OR "older*" OR "geriatric" OR "senior"

AND

"chat bot*" OR "chat-bot*" OR "chatbot*" OR "robot*" OR "avatar*" OR "large language model" OR "artificial intelligence" OR “digital human” OR “virtual human” OR "conversation* agent" OR "AI agent" OR "virtual agent" OR "digital* agent" OR "voice agent" OR "chat agent" OR "relation* agent" OR "companion* agent" OR "comput* agent" OR "conversation* assistant" OR "AI assistant" OR "virtual assistant" OR "digital* assistant" OR "voice assistant" OR "chat assistant" OR "relation* assistant" OR "companion* assistant" OR "comput* assistant" OR "dialog* system" OR "natural language interface" OR "conversation* interface"

AND

"lonel*" OR "isolat*" OR "social exclusion" OR "social particip*" OR "social connect*" OR "social segregation" OR "social companion*" OR "social relation*" OR "depress*" OR "anxi*"

**・IEEE Xplore** Filter: Document title/AB

(Search Terms)

"aged" OR "elderly" OR "older" OR "geriatric" OR "senior"

AND

"chat bot" OR "chatbot" OR "robot" OR "avatar" OR "conversational" OR "AI" OR "language" OR "virtual " OR "digital " OR "voice" OR "chat"

AND

"lonely" OR "loneliness" OR "isolat*" OR "depress*" OR "anxi*"

**・ACM Digital Library**Filter: none

(Search Terms)

(Title:("aged" OR "elderly" OR "older" OR "geriatric" OR "senior") OR Abstract:("aged" OR "elderly" OR "older" OR "geriatric" OR "senior"))

AND

(Title:("chat bot" OR "chatbot" OR "robot" OR "avatar" OR "conversational" OR "AI" OR "language" OR "virtual " OR "digital " OR "voice" OR "chat") OR Abstract:("chat bot" OR "chatbot" OR "robot" OR "avatar" OR "conversational" OR "AI" OR "language" OR "virtual " OR "digital " OR "voice" OR "chat"))

AND

(Title:("lonely" OR "loneliness" OR "isolat*" OR "depress*" OR "anxi*") OR Abstract:("lonely" OR "loneliness" OR "isolat*" OR "depress*" OR "anxi*"))

**Japanese Databases**

**・CiNii Research**Filter: 原著論文限定

**・the National Diet Library**Filter: 原著論文限定

(Search Terms)

"高齢*" OR " 老人"

AND

"chat bot*" OR "chat-bot*" OR "chatbot*" OR "チャットボット” OR "robot*" OR “ロボット” OR "アバター" OR "large language model" OR “大規模言語モデル” OR "artificial intelligence" OR “人工知能” OR "バーチャル" OR "AIエージェント" OR "AIアシスタント" OR "対話システム"

AND

"孤独*" OR "孤立*" OR "うつ" OR "鬱" OR “不安”

**・Ichushi-Web** Filter: 日本語限定、原著論文限定

(Search Terms)

"高齢"/AL or " 老人"/AL

AND

"chat bot"/AL or "chat-bot"/AL or ("生成的人工知能"/TH or "chatbot"/AL) or ("生成的人工知能"/TH or "チャットボット"/AL) or "robot"/AL or ("ロボット"/TH or "ロボット"/AL) or ("アバター"/TH or "アバター"/AL) or "large language model"/AL or "大規模言語モデル"/AL or ("人工知能"/TH or "artificial intelligence"/AL) or ("人工知能"/TH or "人工知能"/AL) or "バーチャル"/AL or "AIエージェント"/AL or "AIアシスタント"/AL or "対話システム"/AL

AND
"孤独*"/AL or "孤立*"/AL or "うつ"/AL or "鬱"/AL or ("不安"/TH or "不安"/AL)


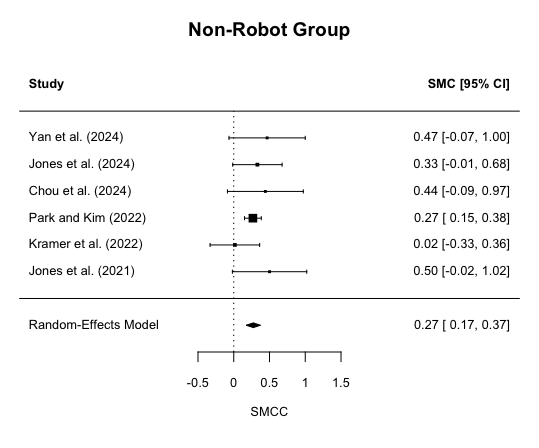
Supplementary Figure S1. Subgroup analysis of standardized mean change using change score (SMCC) for loneliness


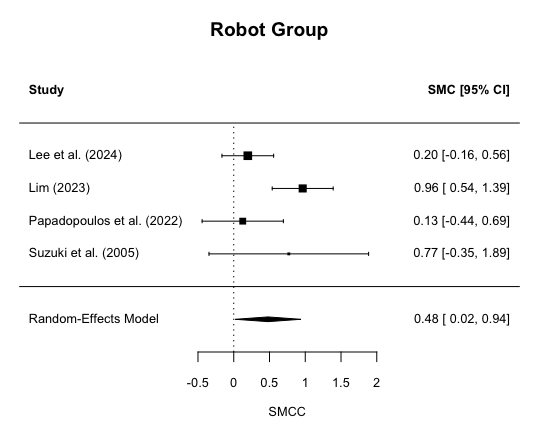


Each study’s point estimate and 95% confidence interval (CI) are shown. The left panel presents results from studies using physically embodied robots, and the right panel includes studies using non-robotic conversational agents. SMCC was calculated by dividing the mean pre–post difference by the standard deviation of the change scores, assuming a pre–post correlation of r = 0.5. Positive values indicate reductions in loneliness following the intervention. Pooled effect sizes for each subgroup were estimated using random-effects models.


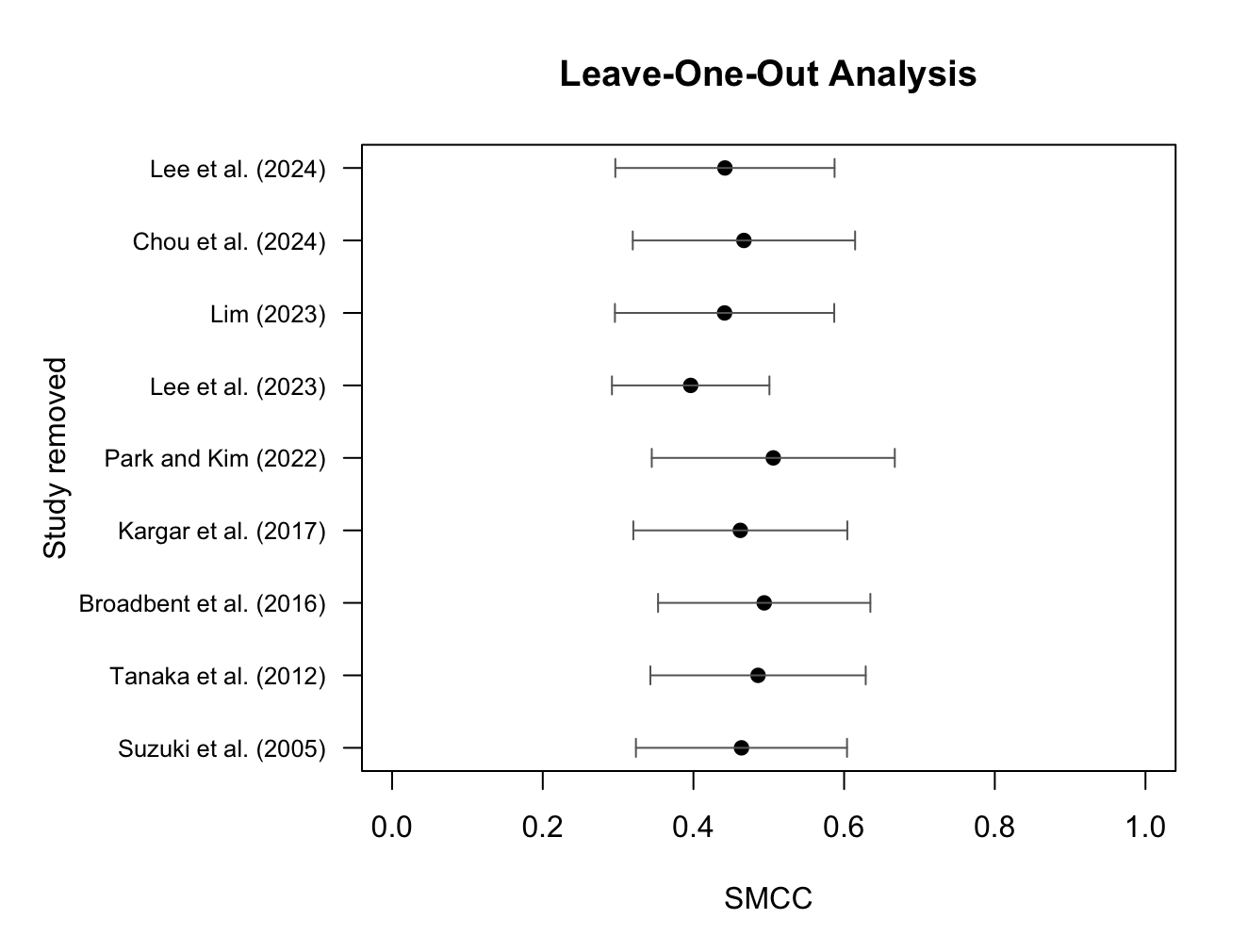

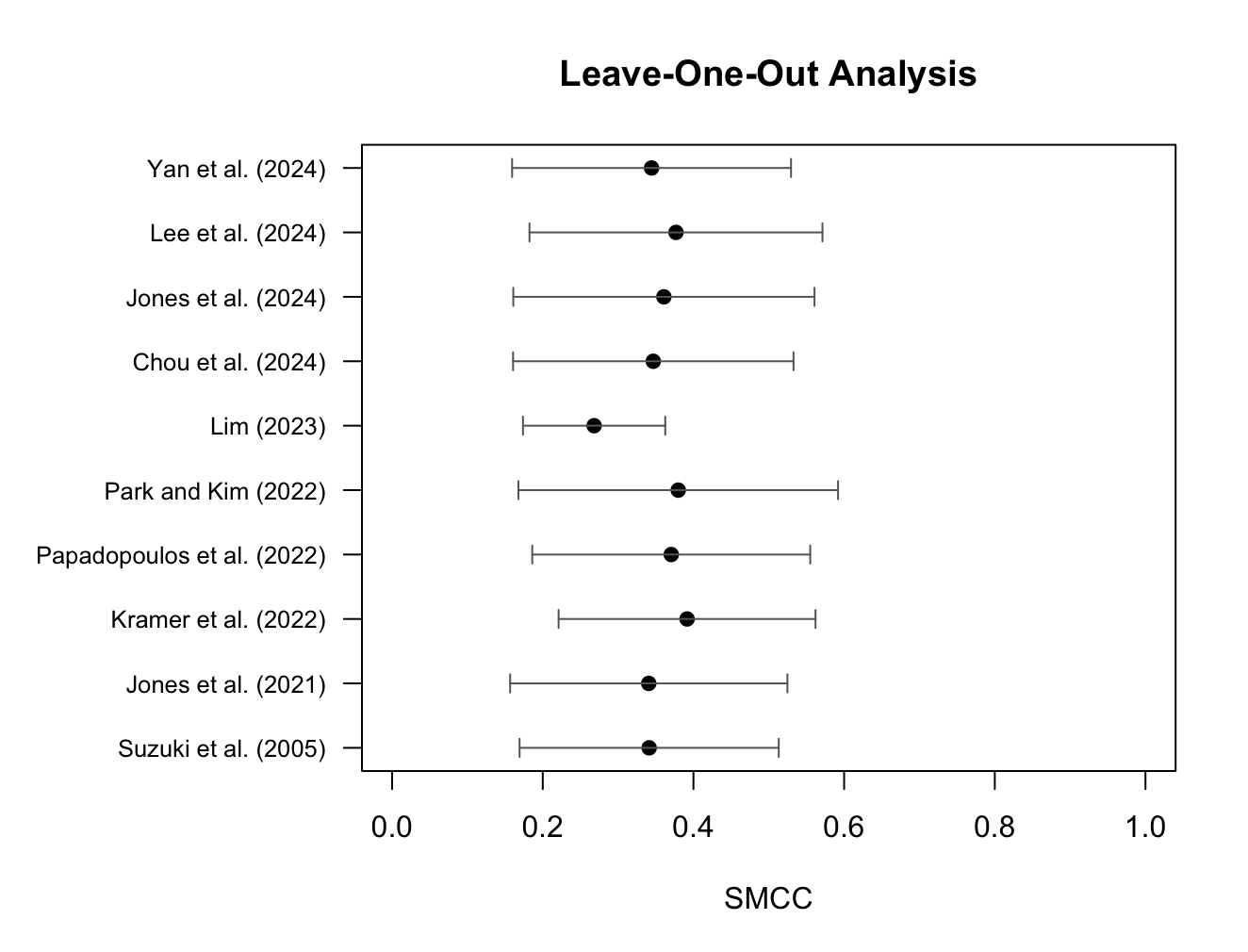
Supplementary Figure S2. Leave-one-out sensitivity analysis for loneliness and depression

Depression

Loneliness

These figures show the changes in the pooled standardized mean change with change score (SMCC) for loneliness and depression when each included study is sequentially removed from the meta-analysis.

Supplementary Figure S3. Sensitivity analysis of pooled SMCC for loneliness under different pre–post correlation assumptions


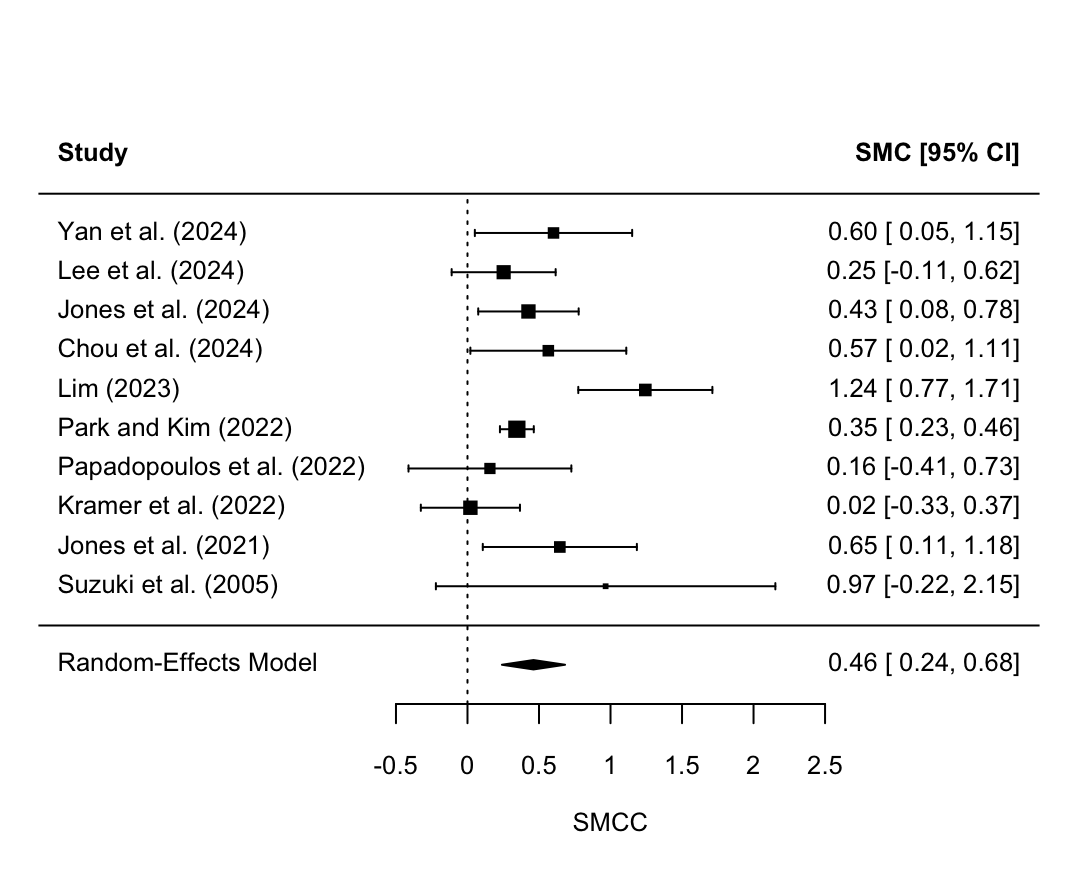

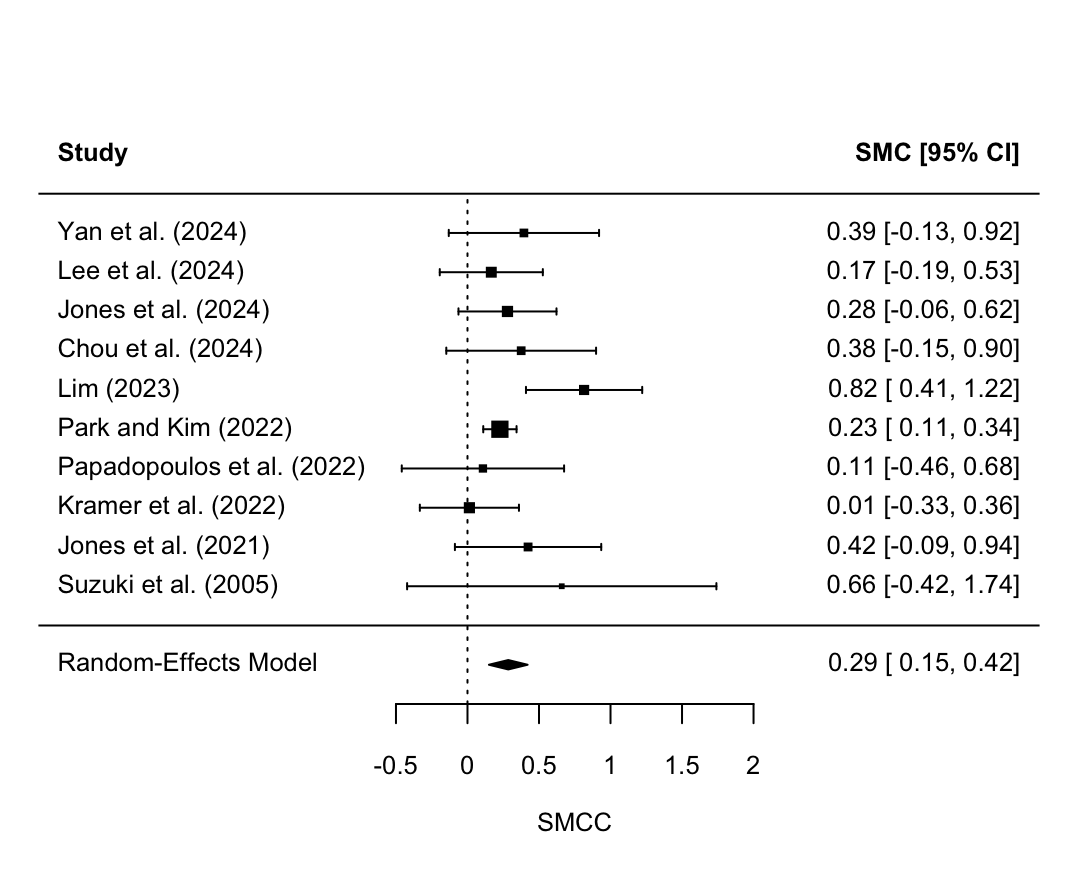


A. r = 0.3

B. r = 0.7

The forest plots show the pooled standardized mean change with change score (SMCC) for loneliness calculated using two different assumed values (r=0.3, 0.7) for the correlation between pre- and post-intervention scores.

Supplementary Figure S4. Sensitivity analysis of pooled SMCC for depression under different pre–post correlation assumptions


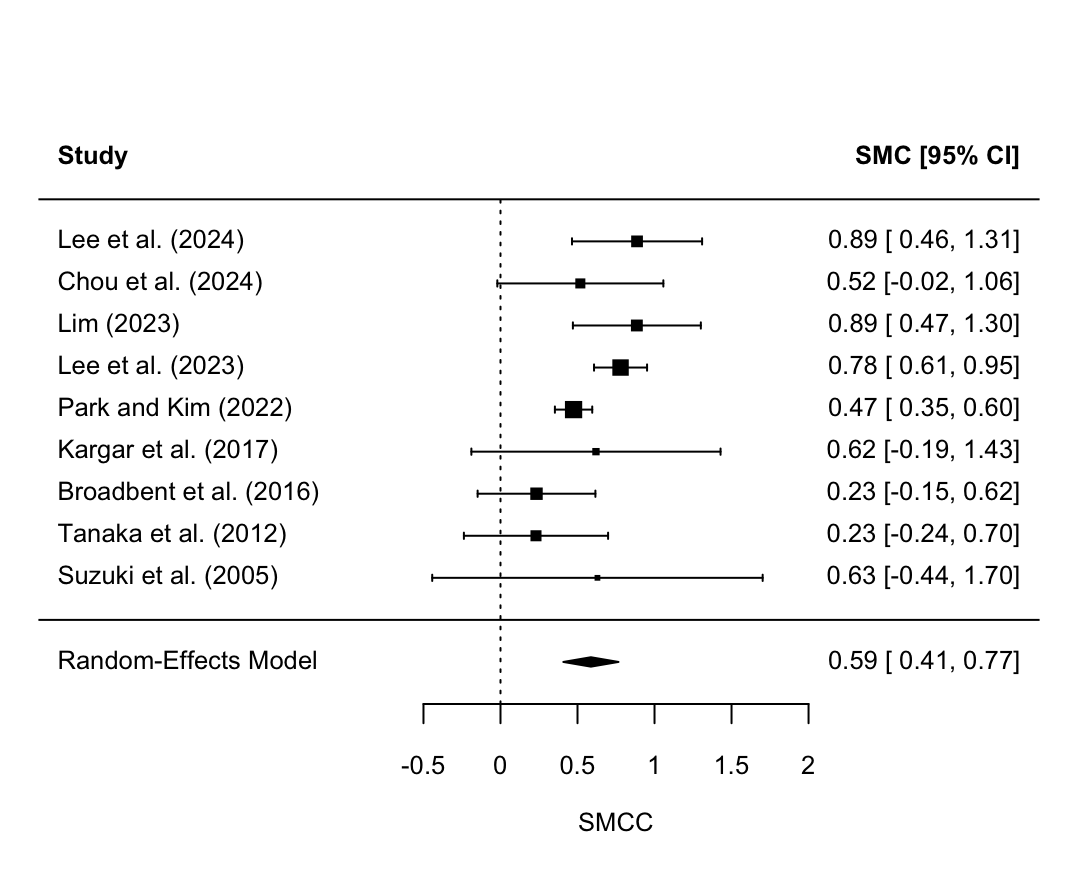

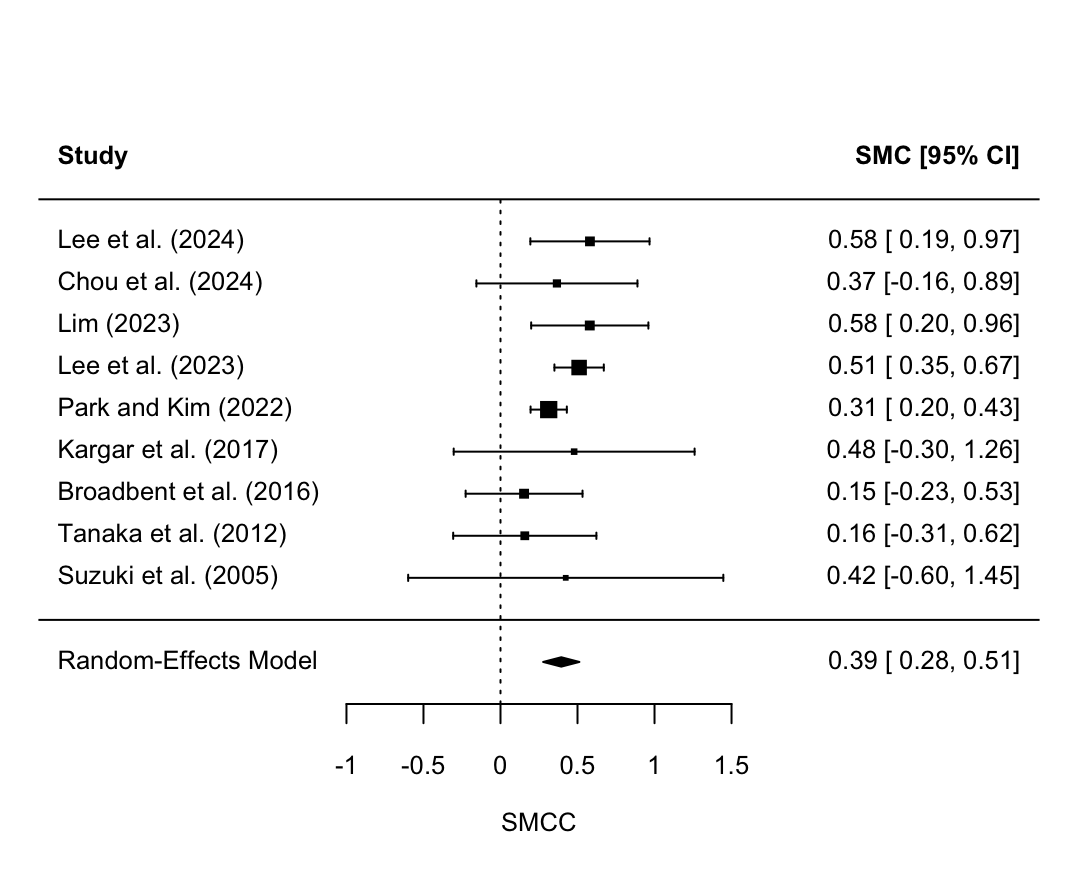


A. r = 0.3

B. r = 0.7

These figures illustrate the robustness of the pooled standardized mean change with change score (SMCC) for depression when varying the assumed correlation between pre- and post-intervention scores.


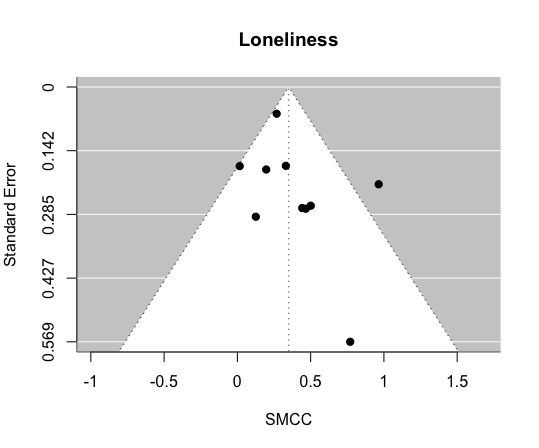

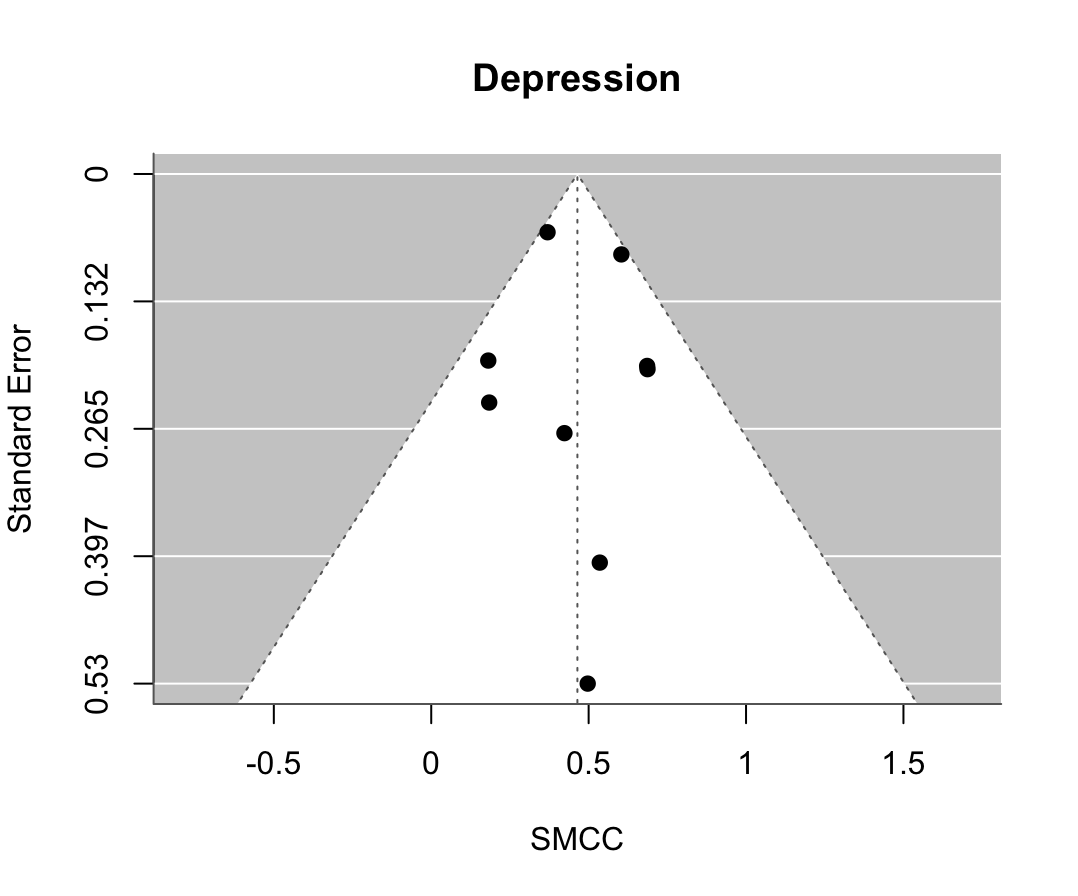
Supplementary Figure S5. Funnel plot of studies reporting loneliness and depression outcomes

Supplementary Figure S6. Modifiable design features of conversational agents.


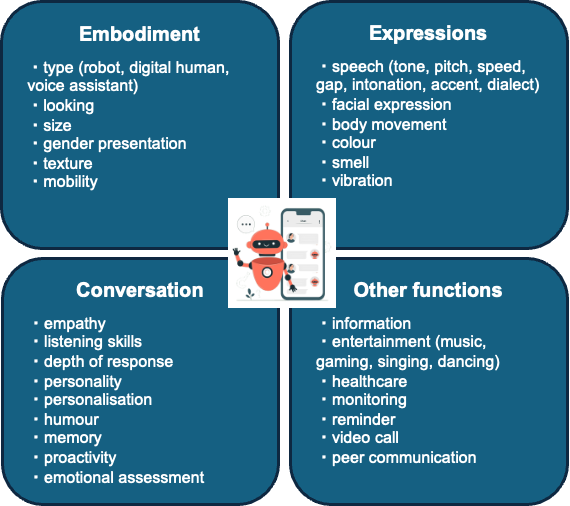


Key design domains of conversational agents include embodiment, expressive capabilities, conversational characteristics, and other interactive functions. Each domain encompasses several modifiable factors that can influence user experience, engagement, and intervention effectiveness. These include physical or virtual appearance (e.g., robot, avatar, voice assistant), speech and non-verbal expressions, personality traits and memory, as well as functionalities such as reminders, entertainment, and video calls.
